# Why does Faithful Epistemic Representation Matter for Management Practices? The Case of the Natural Environment in Management Theory

**DOI:** 10.1007/s40926-022-00220-5

**Published:** 2023-02-06

**Authors:** Rose Hiquet, Claire Wordley, Shahzad Ansari

**Affiliations:** 1 Swiss National Science Foundation, Cambridge Judge Business School, University of Cambridge, Trumpington Street, Cambridge, CB2 1AG UK; 2 Conservation Research Institute, University of Cambridge, David Attenborough Building, Pembroke Street, Cambridge, CB2 3QZ UK; 3 Present Address: Cambridge Judge Business School, University of Cambridge, Trumpington Street, Cambridge, CB2 1AG UK

**Keywords:** Epistemic representation, Management practices, Climate change, Ecosystems services

## Abstract

Management theory is a diverse field where multiple theoretical perspectives coexist and coevolve, leading to conceptual pluralism. While conceptual pluralism is useful for grasping different aspects of the complex reality we live in, it may limit the further development of knowledge on elemental concepts. In this article, we focus on knowledge on the natural environment (NE) in management theory. We argue that management scholars and practitioners often rely on theoretical lenses that tend to reify the NE, thereby limiting the conceptualization of some of the essential properties of the NE. Drawing on the example of the conceptual development of the ecosystem services (ES) at the intersection of economics and biology, we identify the advantages and the limits of interdisciplinary theory-building and testing. Finally, we discuss how tools from the philosophy of science can be useful for proposing a way forward for integrating reliable knowledge on the natural environment in management theory.

## Introduction

Looking back at decades of theoretical crafting in management theory, we face significant silence when it comes to the conceptualization of the natural environment (NE). Management theorists have significantly contributed to a better perception of issues related to the NE, but overall some of its essential characteristics have been left rather unattended. This is problematic since one of the aims of management theory and practice is to tackle grand challenges in society (George et al. 2016), such as helping organizations address climate change (Scherer and Voegtlin 2020). Insufficient knowledge on the NE considerably hinders these aims of management scholars to make meaningful contributions to understanding the cultural and political dimensions of this unprecedented challenge (Ansari et al. 2013). Similarly, it also impact managers who have to rely on their own interpretation of the issue (Sharma 2000), or on the translation of the issue within the corporate context (Wright, & Nyberg 2017).

Despite efforts to craft novel theory for addressing issues related to the NE (Aragón-Correa & Sharma 2003; Bansal et al. 2018; Donaldson 2012; Hahn et al. 2014; Hart 1995; Jennings & Zandbergen 1995; King 1995; Mena et al. 2016; Mitchell et al. 2016; Newton 2002; Peredo & Chrisman 2006; Shrivastava 1995a; Starik & Rands 1995; Starkey & Crane 2003) and even several calls to shift the current paradigm in order to avoid ecological and social disasters (Gladwin et al. 1995; Purser et al. 1995; Shrivastava 1995b), management scholars and practitioners remain ill-equipped for evaluating the implications of climate change and acting accordingly. Despite a few attempts to leverage management theories for studying the topic of climate change (e.g., Ansari et al. 2011), we argue that the roots of this hurdle lie in the under-conceptualization of the NE in management theory.

Conversely, scholars in other disciplines outside management have taken a different stance by integrating knowledge on the NE from a wide range of disciplines, for instance by drawing on insights from the natural sciences. By doing so, they have contributed to the development of actionable theoretical concepts, such as the ecosystem services (ES) concept, at the intersection of economics and biology.

In this article, we aim to understand how an influential theory outside management has been crafted in ways that allow the creation and assimilation of knowledge on the NE but one that has hardly received any attention from management scholars and practitioners. Our analysis, in turn, aims to inform the following questions: To what extent management theories consider NE’s implications for managerial practice in the context of climate change? Which theoretical lenses are more or less useful to tackle the complex problems related to the NE?

First, we review the theoretical lenses that have been used to conceptualize the NE in management theory and their corresponding prevailing applications. This leads us to propose a problematizing review with the aim of understanding whether management theories consider NE’s implications for managerial practice. We explore why the NE remains underrepresented in many management theories and why management scholars and practitioners may be ill-equipped to confront critical issues such as climate change. Then we analyze how theorists from various disciplines can contribute to building on concepts from the natural sciences into a new theory to address climate change by focusing on the case of the ecosystems services (ES) concept. The ES concept has gained considerable momentum since its introduction in the 1970s (Droste et al. 2018), whereas the concepts of *sustaincentrism* and *ecocentrism* proposed by management scholars have remained relatively stagnant. Finally, we draw on the interpretational account of epistemic representation (Contessa 2007, 2014) in order to propose a way forward for a more useful conceptualization of the NE in management theory and practice.

## The Natural Environment in Management Theory

In order to assess critically the concept of the NE in management theory, we followed the principles of the problematizing review (Alvesson and Sandberg 2020). The problematizing review allows the researcher to “identify, articulate and challenge problematic, taken-for-granted assumptions” (Alvesson and Sandberg 2020:1300, citing Davis 1971). Accordingly, we first identified and performed a deep read of the foundational texts related to the NE in management theory.^1^ Then we included representative texts in the review, namely applications derived from the prevailing management theories. This led us to notice which assumptions about the NE can be problematic. Table 1 presents the different problematic assumptions, summarized for each theoretical perspectives.

**Table 1:** The NE in Management Theory

Theoretical lens	Foundational texts	Definition/Conceptualization of the NE	Representative empirical studies	Problematic assumptions
Institutional view	Jennings & Zandbergen 1995	- Ecological system	Hoffman 1999	The assimilative capacity of the ecosphere is functioning (what happens when damage to this capacity is done in an irreversible way?) Organizations are dealing with pressures that come from their environment (for instance institutions or competitors). What happen when pressures come from non-human changes? Changes that have not yet be acted upon by other institutions
Resource-based view	Hart 1995	- Source of strategic capabilities	Russo & Fouts 1997	Performance indicators are only relevant/applicable in a stable context (within the limits of the regenerative capacity of the earth, what happen when we go beyond these limits?) Competition is a key concept (whereas resources might be better preserved when organizations cooperate)
Stakeholder view	Mitchell et al. 1997	- Factor of happiness, of social welfare - Stakeholder	Christmann 2004	The NE is considered only if it is as an object of care/concern for salient stakeholders
Framing perspective	Gray et al. 2015 Hahn et al. 2014 Mena et al. 2016	- Source of concern	Sharma 2000; Wright & Nyberg 2017; Ansari et al. 2013	The pluralistic conceptualization of the NE is harmless
System perspective	King 1995 Starik & Rands 1995	- System	Bansal et al. 2021	The view of an organization as a close system

We complemented our review by studying the literature on climate change and sustainability. A commonality between these two topics in organization and management studies is that they tend to narrow the attention of scholars and practitioners to certain facets of the NE and are generally already integrated into existing frameworks. For instance, climate change is mostly perceived as an issue (Bansal et al. 2018; Härtel & Pearman 2010; Slawinski & Bansal 2015) and treated accordingly in the management literature. Sustainability is slightly different, as management scholars have adopted different approaches to sustainability. For instance, sustainability has been assimilated with normative pressures (Durand et al. 2019), companies’ activities (Crilly et al. 2016; Hahn et al. 2014) and issues (Bansal et al. 2018). These examples show the fragmentation of knowledge on the NE in management theory in part due to conceptual plurality.

The articles in our review either use a single perspective on the NE or multiple perspectives that, at times, are integrated. We propose a review of the theoretical lenses that cover most of the conceptual work that has been done around the topic of the NE in management theory.

We found that the NE has been conceptualized differently in the various theoretical perspectives in management theory. Table 1 lists these different theoretical perspectives and the prevailing conceptualizations of the NE for each of these perspectives.

### The Institutional Lens

One illustrative example of what the institutional approach to the NE looks like was given by Jennings and Zandbergen in 1995. In order to propose testable hypotheses for the study of ecologically sustainable organizations, the authors first undertook a review of organization theory and ecology. Consequently, their conceptualization of the NE is drawn from an ecosystem approach, whereby the NE is reducible to an ecological system, the ecosphere. In their description, there is a clear distinction between the ecosphere (an ecological system) and the sociosphere (a social system) that coexist in the biosphere. The ecosphere has a carrying capacity that provides natural resources for the sociosphere. Once the resources have been used in the sociosphere, they are sent back to the ecosphere in the form of waste that is absorbed thanks to the assimilative capacity of the ecosphere. The main problematic assumption that stem from this lens is that organizations change by reacting to pressures from other social actors (Hoffman 1999). There is no mention of what happen when pressures come from non-human entities. Going back to the conceptualization of the ecosphere, it is for instance unclear how an irreversible damage to the assimilative capacity of the ecosphere is envisioned.

### The Resource-based View

In the same Academy of Management Review (AMR) special issue, Hart (1995) proposed a natural resource-based view of the firm, in which he laid out several principles for “incorporating the challenge of the natural environment into strategic management” (Hart 1995: 998). He started by enumerating various negative impacts of the intense human activity in recent history on the NE. Then he gave examples of negative impacts on the NE that turn into crises for humans, and he depicted an alarming situation for the future. He explicitly pointed out that if nothing was done to change the economic activity, we were risking “irreversible damage to the planet’s basic ecological systems” (Hart 1995: 991). He thus defined nature as ecosystems and subsequently described how three environmental strategies could be used by firms in order to maintain and develop sustained competitive advantage. Accordingly, the representative studies that used the natural RBV have adopted the tenets of strategic management. The main problem with this approach is the overreliance on the concept of competition whereas cooperation might be indeed key in order to preserve the natural resources (Dietz et al 2003).

### The Stakeholder View

The stakeholder perspective does not provide us with a unique conceptualization of the NE. Instead, we are confronted with two standpoints. The first approach is to consider the NE as a stakeholder in its own right (Mitchell et al. 1997; Starik 1993). By doing this, stakeholder scholars recognized that the NE is an entity with various attributes and that it has a direct relationship with the organization. The second approach has been to conceptualize the NE as an element of care and/or concern for stakeholders. In this approach, it is the stakeholders of the organization who have a relationship with the NE, but not the organization itself. This latest approach is prevailing in the stakeholder literature. The main issue with this approach is that not all stakeholders receive the same level of attention from the corporation. Some stakeholders are more salient and thus their requests are more likely to be taken into consideration (Dorobantu et al 2017; Hall et al 2015).

### The Framing Perspective

Framing has been used as a theoretical construct in management and organizational theory at the micro level, the meso level, and the macro level of analysis (Cornelissen & Werner 2014; Gray et al. 2015). The micro level of analysis in the framing literature relates to cognitive processes that are prompted by “priming,” thereby distinct from the social interactionist approach used in the meso- and macro-level analyses (Cornelissen & Werner 2014). The theory that has been developed at the micro level indicates two main conceptualizations of the NE. In some cases, the references to the NE limit its understanding to the environmental outcomes perceived by managers (Hahn et al. 2014). In other cases, the definition of the NE is drawn from a systemic view of nature, which is put forth by ecoscientists (Starkey & Crane 2003). At the meso level of analysis, which is the organizational level, the framing perspective is used to study how meaning is socially constructed and negotiated between organizational actors (Howard-Grenville & Hoffman 2003; Meyer et al. 2016). Accordingly, conceptual investigation can inform us on how the conceptualization of the NE is influenced by the interactions between organizational members (Mena et al. 2016). Noteworthy examples are “greenwashing” (Delmas & Burbano 2011) and the translation of climate change into “business as usual” (Wright & Nyberg 2017). The macro level of analysis concerns cultural frames that are beyond the boundaries of organizations (field frames and institutional frames) and the contestation and/or alignment of these frames (Cornelissen & Werner 2014). The theoretical work from this stream of literature helps us understand how the opposition between field frames contributes to structuring institutional fields (Ansari et al. 2013; Gray et al. 2015). Therefore, it allows to encompass various conceptualizations of the NE, we are in presence of a pluralistic notion of knowledge construction (Eriksson 2004) The main problematic assumption lies in the possibility for different actors to define various conceptualizations of the NE. When several conceptualizations coexist, the risk of misrepresentation increases, namely in the case of framing, the NE has been framed freely from the constraints of physical laws.

### The System Perspective

The system perspective has been applied in different ways in management theory. One approach has been to consider that the connections between the organization and the NE can be analyzed with elements of open systems (Starik & Rands 1995). This analysis presents the NE as a provider of resources (inputs) that are used by the organization. The NE is also giving “feedback” on the use of the resources. This feedback can be positive or negative. Another approach has been to conceptualize the NE as a system, thereby considering some of the characteristics that define it as a system: “multiple equilibria, nonlinearity, irreversibility, and information delay” (King 1995: 965). This perspective appears to be the more suitable, from an epistemological point of view, to a conceptualization of the NE. The challenging part lies in applying the tenets of this perspective practically.

### The Limits to the Current Managerial View on the Natural Environment

Our examination of the various management theories confirms the claim of Shrivastava and Hart (1992) that one central limitation of management studies lies in their narrow view of the organizational environments. For instance, the two theoretical articles that have received the most attention from scholars in the past 30 years^2^ is the article by Hart (1995), and the article by Aragón-Correa and Sharma (2003). Both articles developed the natural RBV of the firm. This view belongs to the environmental management perspective. One of the main problems with this view lies in the fact that the NE is understood solely in terms of resources that are economically valuable. It means that this view limits the understanding of the advantages that organizations get from the NE to purely economic advantages, usually short term and measurable by the existing mean of accounting. It does not allow for the measurement of other types of benefits, such as the benefits related to the preservation of an ecosystem that provides a healthy environment for workers, and therefore makes them less likely to suffer from health problems.

Comparatively, the views belonging to the ecocentric perspective have been underdeveloped. Therefore, there is an unbalanced use of the environmental management perspective and the ecocentric perspective in the management literature.

Another problem that goes beyond the disbalance between stances—and which is related to the lack of understanding of the NE—is that failing to consider the NE appropriately may lead to dangerously illogical reasoning. This type of flaw is illustrated by numerous examples of deficient organizational attention that ultimately result in failures, at the organizational level and beyond (Bansal et al. 2018). In other words, and in the context of climate change that we are considering in this article, what role can organizations play to mitigate the challenges in the face of a climate change catastrophe?

We have also noticed a problematic use of metaphors from natural sciences and conceptual “cherry picking” (e.g., the term “ecosystem and evolutionary theory” (Kapoor & Agarwal 2017)). This practice is harmful, as analogies drawn from another field can only partially help in explaining a complex phenomenon.

Despite the advantages provided by a diversity of disciplinary approaches (Fabian 2000), we see how a minimal level of consensus about an elemental concept can severely limit the development of knowledge for an entire discipline and bears theoretical and practical consequences for scholars and practitioners. According to our review, the main problem about the knowledge on the NE in management theory is the significant absence of a common definition of the NE and otherwise its poor conceptualization, which result in a lack of understanding of the essential properties of the NE.

Much of the theory-building in management has until now contributed to the idea that success in managing organizations can go hand-in-hand with the emancipation of organizations from their biophysical environment. Management scholars are thus now facing the problem of integrating adequate knowledge on the NE in order to be able to address the issue of climate change.

## Interdisciplinary Theory-Building on the Natural Environment

Looking at how an influential theory outside management has developed by integrating knowledge on the NE thanks to the combined work of scholars from different disciplines offers several lessons. That is why we focus on the case of the ecosystems services (ES) research at the intersection of economics and biology. We first present the ES concept before detailing strengths and weaknesses of the ES theory.

### The Ecosystems Services (ES) Concept

**Fig. 1:**
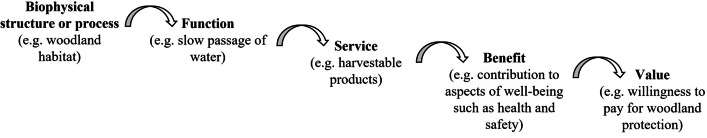
The Cascade Model (Adapted from Potschin-Young et al. 2018)

ES are defined as “the conditions and processes through which natural ecosystems, and the species that make them up, sustain and fulfil human life” (Daily 1997). The ecosystem functions that benefit people (e.g., carbon capture and flood reduction) are nature’s or the ecosystem’s “services” to people, they are often illustrated through the cascade framework in Fig. 1 (de Groot 1987; Ehrlich & Ehrlich 1981; Kellert 1984; Pimentel 1980; Thibodeau & Ostro 1981; Westman 1977).

### Strong Elements of the ES Theory

1. **Integrates human and environmental dimensions.** One of the strengths of the ES concept is that it integrates both human and non-human dimensions. While many management and economic theories have focused solely on humanity, and many ecological theories have focused on non-human nature, ES integrates both elements. The failure of traditional conservation measures—such as protected areas—to halt the destruction of the natural world can be interpreted as a failure of conservation to grapple with the political and economic drivers of damage to the NE (Gómez-Baggethun & Ruiz-Pérez 2011). To conserve the NE—and us humans, who depend on it—theories must successfully integrate human and non-human dimensions. For example, many protected areas (a traditional tool to conserve the NE) are surrounded by land or sea used in a way that is ecologically unsustainable, in so far as it is devoted to economic development and growth (Gómez-Baggethun & Ruiz-Pérez 2011). This mirrors the Western ontological position of humans and nature as operating in separate spheres, and of some natural areas being “allowed” to remain by dominant market forces (Gómez-Baggethun & Ruiz-Pérez 2011). ES allows a different framing, as one where nature is necessary for human societies and markets to exist in the first place (Gómez-Baggethun & Ruiz-Pérez 2011). Some ES work uses a framing of separate ecospheres and sociospheres, or distinct human and natural elements. Other ES work, especially the broader Nature’s Contributions to People (NCP) definition, allows for the framing of humanity as a part of nature (such as in the MA, and IPBES Global Assessment 2019). It is a major step forward to explicitly include human and non-human nature in the same theory, as this paves the way to addressing environmental issues that are both caused by humans and will impact them.
2. **Makes it clear that human life depends on the biosphere.** A related strength of the ES concept is that it makes it clear that humans rely on the NE for their very existence. While this is an uncontested truth in disciplines such as biology and geography, in other subjects scholars might be less aware of the fact that without the complex, interconnected web of life on this planet, no humans could exist. Ecological and paleo-ecological studies show us that without plants, we would not have oxygen to breathe and our climate would be far more extreme, while animals, bacteria, fungi, and other life forms play important roles in regulating ecosystems and nutrient flows. Damaging the NE too heavily could lead to a mass extinction event, which would make human existence far more difficult and painful, if not impossible.
3. **Makes explicit the value of ES not initially captured by the market.** Another strength of the ES theory is that it brings into view the importance of “externalities” that are often not considered in management or economic theories. Externalities are a consequence of an industrial or commercial activity that affects other parties without this being reflected in market prices. For example, the price of dealing with the consequences of climate breakdown is not reflected in the price of gasoline at the pump or in airplane fares. By attempting to integrate these costs and benefits, ES theory attempts to create a financial system that better reflects ecological realities.
  In neoclassical economics, the failure of markets to internalize environmental externalities, along with free riders taking advantage of the public-good nature of ES, is blamed for damage to the NE (Van Hecken & Bastiaensen 2010). Hence, the ES theory calls for the internalization of the NE into markets for ES (Van Hecken & Bastiaensen 2010). Costanza and co-authors (1997) give the example of a coral reef, whose economic value may only show up in commercial fisheries, ignoring its value for recreation and conservation. By paying for recreation and conservation “services,” humans give the reef a “value” beyond fishing. Another example is that of carbon dioxide pollution—an ES approach is to make polluters pay for the damage that they are doing to the NE and society by emitting carbon dioxide in their operations, which will hopefully both drive a switch to using renewable energy and raise revenue to conserve, for example, areas of forest.
4. **Interdisciplinary, testable, and actionable.** The ES concept has engaged with ecology, conservation, climate science in some instances, and economics, making it an interdisciplinary theory. This means it both has inputs from multiple fields and impacts multiple fields. The MA and the 2019 Global Assessment using the concept of NCP also attempt to better integrate social science, public health research, and policy research into the ES theory (Díaz et al. 2018; Reid and Mooney 2016). This interdisciplinarity is considered critical to the success of the related ES and NCP concepts, but also brought many challenges in creating the MA due to different conceptual frameworks between disciplines (Reid and Mooney 2016). For example, natural scientists referred to “drivers of change” in systems, such as saying that beef consumption was driving deforestation, a framework not familiar to economists. It was difficult to recruit social scientists to chapters in the “Current State and Trends” Working Group because it was structured by specific ecosystems and ES, while social scientists identified not with that framework but with how their fields defined the problems being addressed, e.g., “poverty alleviation” (Reid and Mooney 2016).
  Another strength of the ES concept is that, by seeking to produce measurable policy and practice outcomes, elements of the theory are testable. We can test whether markets in carbon reduce carbon pollution; whether payments for ES lead to the retention of forests and other ecosystems; whether people are more likely to conserve nature if they are told its financial worth; and so on. This testability is itself a strength of the concept, as it facilitates the adoption of elements which work well, the removal of parts that work badly, and the improvement of both the theory and the way it is implemented.

The ES concept has also transcended theory to be used by practitioners – governments, businesses, and NGOs.

“Over a period of about 15 years, an eye-opening metaphor intended to awaken society to think more deeply about the importance of nature and its destruction through excessive energy and material consumption transformed into a dominant model for environmental policy and management in developing countries and for the globe as a whole”(Norgaard 2010: 1219).

Over the past three decades, an increasing number of ecosystem functions have been classified as services, been valued in financial terms, and been incorporated into payments for ES and markets for ES schemes (Gómez-Baggethun et al. 2010). It is likely that the focus on valuing ecosystems in financial terms, and in incorporating nature into the dominant ideology of market-based solutions, has amplified the appeal of this concept and contributed toward its uptake into policy (Gómez-Baggethun et al. 2010). The MA alone contributed much to the “mainstreaming” of the ES concept. The MA was primarily intended as a resource for governments and the private sector (Reid and Mooney 2016). The evaluation of the MA in 2006 concluded that there was “widespread evidence that the assessment is having an impact on the intended audiences, but the extent of that impact is very mixed, with some institutions, regions, countries, and sectors significantly influenced by the MA while others have not been influenced at all” (Wells et al. 2006).

5) **Open to various forms of knowledge.** The Intergovernmental Science-Policy Platform on Biodiversity and Ecosystem Services (IPBES), designed to assess the state of the world’s biodiversity and ES, was developed after the climate equivalent, the IPCC, had been established for a while. This allowed the avoidance of perceived failings in how the IPCC functions, including limited participation from the Global South and from Indigenous communities, and a failure to meaningfully integrate diverse perspectives from different disciplines and from outside academia (Montana 2017).

As a result, IPBES has actively sought ways to incorporate Indigenous and local knowledge into its work. This has included some of the landmark publications using the ES concept, for example the Pollinator Assessment and the IPBES 2019 Global Assessment (Díaz et al. 2018; Montana 2017; Reid and Mooney 2016; Tengö et al. 2017). Indigenous and local knowledge (ILK) about ecosystems and environmental change is a valuable but often overlooked source of information, as it is not gathered or presented in the same framework as scientific knowledge. ILK is often the only information available for specific sites, and can add a historical dimension (such as how migration patterns of animals have changed in South America). This can help to inform local and regional decision-making (Reid and Mooney 2016). Furthermore, including Indigenous Peoples in assessments helps to ensure that issues of relevance to Indigenous Peoples are included, that their worldviews and values are represented, that challenges such as how climate change may affect Indigenous Peoples specifically are confronted, and that Indigenous knowledge about how they responded to previous environmental challenges is documented (Reid and Mooney 2016; Tengö et al. 2017). Indigenous knowledge is based on continuous observation over long time periods and framed through a distinct set of worldviews, which can complement scientific knowledge but can also be challenging to incorporate alongside it (Montana 2017; Tengö et al. 2017).

IPBES assessments have developed processes to include diverse knowledge systems through the production of typologies or lists of comparable options, allowing a range of knowledge systems to coexist (Montana 2017). These typologies are “a solution to create an agreement out of disagreement, to create a consensus out of dissensus” (Borie and Hulme 2015: 494). They include typologies of knowledge systems, such as scientific, Indigenous, and local; typologies of values, such as anthropocentric and non-anthropocentric; and different valuation methods, such as ecological, cultural, economic, indigenous, and public health (Montana 2017). Including the diversity of values and valuations around the NE is especially important, as values frame the responses to all discussions. IPBES is currently reviewing the range of values and valuation methods around the NE.^3^

Several other studies using the ES framework have also incorporated Indigenous knowledge, showing that it can be done, although they remain a minority. For example, Harmsworth and Awatere (2013) built a framework for discussing ES that is based on Māori knowledge, values, and perspectives. This makes important distinctions between “cultural values” and “cultural services” and includes the concept that all “benefits” are reciprocal rather than one way (e.g., *from* the NE *to* humans). Taking a different tack, Ouédraogo and co-authors (2014) used interviews to identify the plant species that provided different ES to people in a village in Burkina Faso, with Cummings and Read (2016) similarly identifying the plants that provided important ES to Indigenous Peoples in the Northern Amazon.

Shaping the ES concept so that it has use and resonance beyond academia is important. While much of the framing of ES has drawn on an economic and neoliberal framing, other ES framings have incorporated diverse worldviews and relationships with the NE.

### Weaknesses of the ES Theory

1. **Use of an instrumental lens has been criticized.** The ES concept has for the most part taken an instrumentalist view of nature—that we should conserve nature because of the value it provides for humanity. Some scholars have stated concerns that the instrumentalist lens does not adequately account for nature’s inherent worth, non-measurable human interactions with nature, human responsibility toward nature, or humans being a part of nature (Díaz et al. 2018). There are concerns that a purely instrumentalist view may offend those who have a different relationship with nature. For example, thinking of nature as a service provider (especially a non-consenting and unremunerated service provider), or of ecosystems as factories, may be anathema to cultures who see nature as intrinsically valuable (Chan et al. 2016; Díaz et al. 2018).
  Some scholars call for the use of an intrinsic lens—the idea of conserving nature for nature’s sake (McCauley 2006). In this framing, while it is acknowledged that humans rely on nature for our survival, there is a moral imperative to conserve other life forms and natural systems because we have no right to destroy other living beings on the only planet we know of that supports life. In this framing, nature is valuable and should not be destroyed, even where specific species or systems provide no specific benefit to humans, or where specific species or systems cause harm to humans. However, Costanza and co-authors (1997) point out that this framing has been used for many years and the NE is still deteriorating.
  Other conservationists have called for more emphasis on relational values—how humans and nature relate with each other (incorporated into NCP). These relational values evoke our rights and responsibilities toward nature, as well as cultural identities and social cohesion (Chan et al. 2016). Chan and co-authors argue that neither intrinsic nor instrumental framings have garnered sufficient public support, but that relational framings may do better. These relational values are present in worldviews such as the Latin American concept of “*Vivir Bien*,” or living in harmony with people and nature. There is little empirical evidence on the effectiveness of relational values messaging, although Klain et al. (2017) find high support for relational statement such as “plants and animals, as part of the interdependent web of life, are like ‘kin’ or family to me, so how we treat them matters.” Some scholars use a mix of intrinsic, instrumental, and relational lenses to make the case for not destroying the NE (Díaz et al. 2018).
2. **Counterproductive effects of financializing nature.** Financializing nature may reduce support for conserving the NE. In empirical psychology, the intrinsic, relational, and instrumental lenses have been explored in terms of “priming” people with different “values” (Crompton et al. 2014). Psychologists identify intrinsic, self-transcendent values (I/ST values) as those of social justice, equality, unity with nature, and self-acceptance (Grouzet et al. 2005; Kasser and Ryan 1996). They identify extrinsic and self-enhancement values (E/SE values) as values of wealth, social status, or public image (Grouzet et al. 2005; Kasser and Ryan 1996). I/ST values are associated with support for both social and environmental causes, while E/SE values are associated with lower levels of concern about social and environmental problems (Crompton and Kasser 2010).
  Notably, in this psychological framework, conserving the NE because of the benefits it provides to humans can come under I/ST values such as universalism (defined as “understanding, appreciation, tolerance, and protection for the welfare of all people and for nature,” Schwartz and Bardi; 2001: 270)—unless the argument is that we must conserve the NE to gain wealth, status, power, or public image. Wanting to conserve the environment so that people in tropical countries are not forced to migrate is an I/ST value. Using financial arguments to explain the need for environmental conservation is seen as activating E/SE values for wealth. Furthermore, research has shown that most people value intrinsic values over extrinsic ones, across a wide range of cultures—universalism, which encompasses care for nature, is among the top three highly ranked values (Schwartz and Bardi 2001).
  Other scholars question whether framing nature within the ES concept may modify the way humans perceive and relate to nature in the long term (Büscher et al. 2012; Kosoy and Corbera 2010). Institutions and organizations can modify human behavioral patterns and motivations. For example, by creating economic incentives for conservation, the market values of individualism and competition (E/SE values) may be fostered in communities and societies previously structured upon community and reciprocity values (I/ST values). Vatn (2010) suggests that financial payments may change the rationale from being what is best to do for the community toward what is individually best to do.
  The empirical psychological work on values activation does not suggest that we should not point out the utility of nature for humans, but does suggest that if we use an instrumental lens, it should not be done in a way that invokes transactional values, or values around wealth and power (Blackmore et al. 2013). Emphasizing the financial value of nature, as tested in recent studies, can lower people’s motivation to act for nature (Crompton et al. 2014) and alter their support for environmental protection (Rode et al. 2017).
3. **Problematic terminology**. Terms such as “ecosystem services” narrow human relationships with the NE. The use of language can activate different values, create resonance, and change our behavior. Language can prime different values, as seen in the previous section. In an experiment, volunteers given a “Consumer Reaction Task” became more competitive, less likely to engage in collective action, conserved less water, and felt less personal responsibility for environmental problems than volunteers given an identical task labeled a “Citizen Reaction Task” (Bauer et al. 2012). The word “consumer” is more linked to E/SE values such as status, wealth, and power, while “citizen” is more linked to I/ST values such as social justice and responsibility. Similarly, participants in “The Wall Street Game” behaved more selfishly and treacherously than those playing the identical “Community Game” (Liberman et al. 2004).
  Words such as ES and “natural capital” have served to define complex phenomenon in a simple manner and to ease the communication between disciplines, especially between the disciplines of ecology and economics (Granek et al. 2010). However, there are criticisms that these terms have limited the conceptualization of the NE. The term ES invokes a transactional relationship where nature is a service provider and humans are consumers (Sullivan 2009). Some anthropologists question the universality of the ES “language,” asking who is creating this language, for whom, and who is excluded from this language (Sullivan 2009). The ES language can be seen to be restructuring human/non-human relationships, displacing and “othering” worldviews that have, in many cases, served to conserve biodiversity over millennia, such as many traditional Indigenous cultures that do not see nature as a service provider or as capital (Sullivan 2009).
  Sullivan (2009) asks what is the “nature of nature” as perceived within an ES/NC framing—and does this framing exclude the many cultures who see a different “nature of nature”? The language of people as “service providers” can reshape people’s perceptions of themselves if they are involved in a PES project; whatever they saw themselves as before, they are now “providing” a “service” for financial gain by realizing a vision of the land conceived, usually, by people living elsewhere (Sullivan 2009). Thus the ES concept and the language used to describe it may narrow people’s unconscious understanding of the NE, the relationship of humans to the NE, and the solutions to limit the damage to the NE.
4. **Buying into ideology**. Since the 1980s, most international policy has been conducted within the ideology of neoliberalism (Harvey 2005). Neoliberalism is a political-economic theory that states that human well-being is best served by increasing market reach and economic growth in an institutional framework of strong property rights, free markets, and free trade. It encompasses practices such as privatization, state use of force to defend private property rights, reduced role of the state in the economic and social spheres, deregulation, and the expansion of markets into areas that were formerly non-commercial or not free markets, such as policing, prisons, the military, health care, education, and the environment (Harvey 2005).
  Neoliberal politics are often credited with reducing environmental protections through deregulation (removing environmental protections and state intervention in cases of environmental destruction), and through a focus on continued economic growth (Büscher et al. 2012; Hickel and Kallis 2020; Jackson 2009). The neoliberal ideology of reducing regulations (often referred to as “red tape”) has led to some governments seeing environmental regulation as a “last resort” (Great Britain. National Audit Office 2014). Citing the cost to businesses, policymakers in the EU, USA, and elsewhere have promoted voluntary approaches such as industry self-regulation and voluntary codes of conduct as an alternative to legislation (Great Britain. National Audit Office 2014). A review on the effectiveness of voluntary approaches found that the majority were missing their targets (McCarthy and Morling 2015).
  As neoliberalism focused on infinite economic growth has become the dominant global ideology, it has been increasingly absorbed into conservation science and practice (Büscher et al. 2012; Sullivan et al. 2014). The neoliberal framing holds that markets will solve all issues, so if there is a problem, better market integration and reduced regulation will help to solve it. Market environmentalism is a predominantly neoliberal framing that aims to conciliate economic growth with environmental conservation. Market environmentalist approaches hold that we need to enfold the NE within markets, either as well as or rather than enact state legislation or community regulation to protect ecosystems and curb pollution.
  However endless economic growth is increasingly considered incompatible with a functioning natural world, as attempts to ‘decouple’ the economy from natural resource use have not reached anywhere near the necessary levels (Jackson 2009; Raworth 2017; Hickel 2019). All economic activity has some natural resource use, and therefore ever-increasing economic growth will lead to ever-increasing impacts on already stressed ecosystems and scarce resources (Jackson 2009; Hickel 2019).
5. **Moral concerns**. Some concerns about the commodification of ecosystems have been more ethical or moral in nature. Some scholars have argued that some things should not be for sale—for example, humans, organs, votes, public office, parliamentary questions, love, friendship, and natural systems (McCauley 2006; Sandel 2012). Of course, physical components of the NE such as food have been on sale for time immemorial—the more contentious issues are whether, why, and how we price ecosystems and wildlife.
  Costanza and co-authors (1997) argue that we are constantly valuing things such as human lives and ecosystems, whether we know it or not. For example, when we set construction standards for bridges, we do so because spending money on construction saves lives. They go on to say that “although ecosystem valuation is certainly difficult and fraught with uncertainties, one choice we do not have is whether or not to do it. Rather, the decisions we make as a society about ecosystems imply valuations (although not necessarily expressed in monetary terms). We can choose to make these valuations explicit or not; we can do them with an explicit acknowledgement of the huge uncertainties involved or not; but as long as we are forced to make choices, we are going through the process of valuation” (1997: 255).
  The difference, for those concerned about the ethics of valuation, seems to lie not in weighing up and valuing different options and their impacts on ecosystems, but in the conversion of these values to monetary ones, with all the exchangeability and equivalence that this implies (Sullivan 2009). For example, when we set construction standards, we do not price human lives in dollars and put those figures into a cost–benefit analysis; we say bridges must not collapse as no human deaths are acceptable, and spend whatever is necessary to achieve that. Valuing ecosystems and their benefits in a non-financial manner may get around this impasse; pricing something is not the same as valuing it, and the two terms should not be used interchangeably.
  Discomfort around financially valuing nature is widespread in the wider public beyond academia. This is apparent from the valuation exercises where people have expressed distress or moral outrage over being asked about their “willingness to pay” for nature (Clark et al. 2000). Participants in some valuation exercises have in some cases rejected the idea of financially valuing nature, and wanted instead a dialogue-based process between local people, scientists, and policymakers (Clark et al. 2000; O’Neill and Spash 2000).
6. **Practical difficulties in applying the concept.** Applying the ES concept has also proved to be challenging on several accounts. Providing an exhaustive list of the practical challenges that have come along the application of the ES concept is beyond the scope of this article so we only detail here three of them: ecosystem disservices, financial practices, unbundling ES for valuation and trade.

A key assumption of the ES concept is that, because humans are dependent upon the NE, actions that protect the NE will automatically benefit humans. However, on examining the ES literature, win-wins (where both people and the NE benefited within the spatio-temporal scale of the study) are the exception rather than the rule, at least in the short to medium term (Howe et al. 2014). In fact, while overall we need the biosphere to survive, at the local scale a particular ecosystem, or part of an ecosystem, can even cause damage or do a “disservice” to people (McCauley 2006). The biosphere, as McCauley points out, does not act in service to any particular components. While we rely on the biosphere for our very existence, it also visits humans with hurricanes, floods, wild animals raiding our crops or killing us, diseases, and more. But breaking the NE down into “good” and “bad,” “valuable” and “non-valuable” parts is, as described before, an ecological impossibility. For one thing, the same species or ecosystem element can be beneficial in some contexts (fruit bats pollinating crops) and negative in another (fruit bats transmitting Ebola). This leads to practical questions about whether and how to account for “disservices” in, for example, PES schemes, as well as wider questions about using the instrumental lens to conserve the NE—should we only conserve the parts that we see bring us benefits? Given the complex interactions and feedbacks between the parts of ecosystems that we see as providing “services” and “disservices,” is it even biologically plausible to do so?

That financial banking practices are based on massive indebtedness, and the proliferation of capital through splitting practices is another source of concern to those who worry about market environmentalist approaches (Sullivan 2014). In finance, banked capital can be transformed to debt and split into tradeable packages to generate value through “securitization” (Sullivan 2014). These models are problematic ones for “natural capital,” the material of the natural world; rivers, forests, elephants, ants, and soil microbes cannot be destroyed now and repaid later, nor can they easily be split into tradeable packages.

Another issue with making the economic case to protect nature is that the economic case can change with shifting markets (Silvertown 2015). This means that solutions that work financially now may not work next year, or even next week. Market changes can reinforce the notion that the ES concept seeks to avoid—that destroying the NE is acceptable if it yields a profit.

To be functional for capital, commodities must be alienable, fungible, and mobile (Robertson 2004). One of the major issues with accounting around ES is that to financially value ES and make them tradeable, we must separate or “unbundle” them from each other and place boundaries over them (Boyd and Banzhaf 2007; Duke et al. 2012). However, in the physical world, ecosystem functions are interconnected and interdependent, and often cannot be separated in a meaningful way (Kosoy and Corbera 2010; Vatn and Bromley 1994). Compressing complex realities into a simple metric of financial worth is likely to result in a non-trivial loss of information (Vatn and Bromley 1994). The NE consists of huge numbers of not always discrete processes and feedback loops maintaining balance as matter and energy flow through it (Vatn 2000). The NE system has evolved over vast time spans in natural “experiments” of trial and error, leading to a self-organized system of mutually supportive processes (Vatn 2000). This makes it difficult if not often meaningless to identify single characteristics of the system to trade. This is further complicated when we attempt to trade in ecosystem services in ways that require physical transformation, transportation, or substitution (i.e., biodiversity offsetting could involve cutting down an ancient woodland in one place to plant new saplings in another; Vatn 2000). Valuing each ecosystem component in a unified manner (generally in currency) creates the appearance of equivalence and commensurability between different aspects and categories of nature and between different locations and times, permitting the process of “offsetting” (Sullivan 2014). But the original functions of the service may be altered or cease if this service is moved or changed.

## Addressing Climate Change in Management Theory

Going back to the different theoretical lenses in management theory, we rely on the example of the ES research concept and on insights from the philosophy of science to help management theorists develop useful ways to tackle the problem of climate change for management practice. We argue that there is a major problem with the way knowledge on the NE has been diffused in management theory. Most of the conceptual work has been done on topics related to NE (e.g., climate change, sustainability) and not primarily on the NE. A direct consequence of this peripheral conceptualization is a partial understanding of the NE and a lack of adequate conceptual tools to fully grasp the complexity of the climate change phenomenon. Furthermore, with the exception of the system perspective, social sciences tend to subjugate the theoretical perspectives that have been used so far, thereby inherently limiting knowledge on the NE.

We resort to philosophy of science because it is a discipline that helps us understand how knowledge is generated by sciences, and there is a long tradition of studying theory-building at the interface of various scientific fields in this discipline (Bechtel 1984; Darden & Maull 1977). The inferential conception of scientific representation is particularly relevant to our endeavor because it provides us with an explanation for misrepresentation (Suárez 2004). Misrepresentation can happen when one takes a deflationary approach to inference, that is to say when a concept is used superficially, without the aim of effectively gaining insight about what the concept entails (Suárez 2015). Misrepresentation of the NE in management theory appears to be at the core of the problem, since it furthers unrealistic accounts of the world. For instance, there is not a single article in the top-tier management journals that provides the reader with a comprehensive explanation of what finite natural resources mean and what are the impacts of this reality for organizations.

We encountered the same shortcoming while looking for articles containing the expression “planetary boundaries.” We found a very limited number of articles in which this expression, which is related to the limits of the biophysical world, was mentioned, despite the fact that this approach has attracted interest from both policymakers and the business sector (Steffen et al. 2015).

We propose a way forward by laying out principles for developing the conceptualization of the NE in management theory. We argue that an “interpretational account” of representation (Contessa 2007) should be preferred over the deflationary approach that currently prevails in the management literature. An inferential approach to representation is deemed “deflationary” when the user is not looking for “deeper features to representation” but is instead considering “the surface features of representation” (Suárez, 2004). In other words, under the deflationary approach, the user is not inferring from the representation. Conversely, when taking on an interpretational approach, the user is able to perform inferences while considering a representation.

Our proposition includes a set of specific epistemic features that could support the theoretical development of the NE in management theory for addressing climate change. The work on epistemic representation and particularly the focus on the representational function of scientific models (Contessa 2007, 2014; Suárez 2004) is helpful for our study. We present the key characteristics of this approach and propose a way to apply it for an adequate integration of knowledge on the NE in management theory.

For clarification “to say that a representation is an epistemic representation is just to say that it is a representation that is used for epistemic purposes” (Contessa 2014: 134). As an example, let’s consider several objects related to the representation of an organization. The logo of the organization would be a denotation, all organization charts (previous and current) would be epistemic representations and the current organization chart would be a faithful epistemic representation. Denotation and epistemic representation are both necessary but insufficient for obtaining a faithful epistemic representation. A faithful epistemic representation is obtained when the users interpret the representation in terms of the system it represents.

More specifically, in his defense of the interpretational account of epistemic representation, Contessa (2014) gives a central role to analytic interpretation. Following up on the previous example, it is only when a user (e.g. a management practitioner) is interpreting the meaning of the organizational chart that it becomes an epistemic representation. The user, being familiar with interpreting other organizational charts, can interpret this chart and thus infers that lines represent links between different organizational units. Accordingly, Contessa proposed that the relation between epistemic representation and valid surrogative reasoning is dependent on the interpretation made by the users.

Our analysis points to the fact that the prevalent type of approach to the representation of the NE in management theory is deflationary. This approach to the knowledge on the NE doesn’t allow scholars and practitioners to infer about the NE. Indeed, except for some management theoretical perspectives (see Sect. 1), the NE has not been part of the representational content of models and when it is, its reification is problematic.

How then can management scholars infer about the NE while looking at a representation of the NE in management theory? We argue that the adequate integration of knowledge on the NE in management theory goes through a faithful epistemic representation of organizations in the NE. We now propose a way forward for moving from a deflationary approach to the possibilities of inferring from an epistemic representation.

A faithful epistemic representation implies a sufficient similarity between the representation and the system it is representing (ie morphism between the structures of the representation and the system represented). Management theory faces here a major problem, because a main source of modelling for the field is classic economic theory where natural resources were initially decoupled from economical thoughts. “The natural resources are infinite […] they are not the object of economic science” (Say 1803). The models that are implying that indefinite linear growth is possible are thus ontologically wrong because they rely on the assumption that there is an indefinite supply of natural resources (Raworth 2017; Hickel 2019). Contrary to relying only on assumptions that stem from artificial constructs, the integration of knowledge on the NE would help to develop management theory for the finite world which we live in. Furthermore this integration would help to include some of the essential parameters of natural eco-systems (eg non-linearity, recursiveness, wholeness) into the epistemic representation of organization in the NE and thus help management scholars and practitioners to perform new surrogative reasoning (Contessa 2007; Swoyer 1991).

The level of necessary similarity is also defined by the purposes of the users (Contessa 2014; Frigg & Nguyen 2017; Giere 2010; Teller 2001). Not all representations need to be completely faithful, but they need to be sufficiently faithful for the purposes of the users (Contessa 2014). This condition explains why the lack of knowledge on the NE has not been considered as a common issue for all management scholars yet (ie models focused on economic performance that had infinite natural resources as an underlying assumption have not been dismissed). The issue of climate change presses management scholars to renew their purposes and think beyond the assumption that economic performance is a sufficient condition for organizational survival and success (Ansari et al. 2011). However the current epistemic representation of a successful organization in management theory inhibits the inferential ability of management scholars because of the absence of sound information on the NE.

## Discussion

Despite an ever-growing body of work on the natural environment (NE) in management literature, the NE has been under-conceptualized in management theory. As a result, both management scholars and practitioners are lacking elemental knowledge on the NE that places limits on how they can face critical issues such as climate change. Furthermore when the NE is referred to in management theory it is mostly done through distinctive theoretical perspectives, therefore limiting a common understanding of the NE. Unlike management scholars, scholars from other fields (mostly economics and biology) have worked with an actionable concept of the NE, the ecosystems services (ES) concept. While the use of this concept present both theoretical and practical advantages we have also described some of its limitations. Leaning on the evolution of this concept and on insights from the philosophy of science we have proposed a way forward for integrating knowledge on the NE in management theory, in order to achieve a more faithful epistemic representation.

The case of the ES concept underlines the many problems that can unfold with inaccurate epistemic representation and the interpretation of a concept by its users. Despite the fact that the ES concept is an accepted epistemic representation of the relations between the ecosystem functions and the economy, not all the users of the concept have been able to perform surrogative inferences about these relations. This limitation stems from the fact that some users don’t interpret the concept in terms of the natural and inseparable relations between the ecosystem functions and the economy. They have instead adopted an interpretation of the concept in other and often dichotomous terms (e.g., either economic or non-economic).

In this article, we have described some risks related to the limited conceptualization of the NE in management theory. Specifically, we have explained how the deflationary approach to the NE in the epistemic representation of successful organizations limits the inferential ability of management scholars and practitioners. For instance, the separation of the social and ecological systems is a common assumption in management theories (Bansal & Song 2017; Jennings & Zandbergen 1995). This separation implies an “impermeability” between social entities and natural entities, which is conceptually useful for simplifying a complex world and understanding some mechanisms. However, it does not allow management scholars and practitioners to perform surrogative inferences from the conceptual model to the actual system of relationships that exist in reality, thereby considerably limiting if not rendering impossible their ability to adequately address critical issues such as climate change.

Management scholars from various perspectives have called for a renewal of the research on organization and the NE (Ansari et al. 2011; Bansal & Gao 2006; Hoffman & Jennings 2015). In this article we have argued that one way to do it is by developing a faithful epistemic representation of the NE in management theory. Looking at the example of the ES concept at the intersection of economics and biology we have presented the advantages and the limits of an actionable and interdisciplinary concept. Insights from the philosophy of science gave us the means to sketch a way forward by considering not only the deflationary account of representation but also the interpretational account of epistemic representation.

### Implications

The question of practical relevance is of high importance to management scholars (Kieser et al. 2015). Our review of management theoretical perspectives on the NE has raised concerns regarding the current and future relevance of our field in the face of the current climate change emergency. In this article, we have proposed to rely on insights from the philosophy of science to help conceptualize the NE. Knowledge on the NE should not be reduced to a sub-field (sustainability). Rather, it should be systematically integrated into all the models that are developed in management theory. This integration could have direct and concrete implications for the various theoretical frameworks which are constitutive of management theory. For instance it could advance the debate on the inclusion of the NE as a stakeholder (Haigh & Griffiths 2009; Waddock 2011) and contribute to solving the problem of the framing of climate change (Campbell et al. 2019; Hoffman 2011).

Furthermore, it could also help managers come to terms with the urgency of adopting economic models that are compatible with the limits of our biophysical environment (e.g. the circular economy), in accordance with the propositions advanced in the recent special issue on ecological management (Blok, 2021).

Finally, management practitioners will not only have more concrete tools at hand to help them addressing the climate emergency, they will also be able to infer from the epistemic representation and thus possibly develop creative solutions for the idiosyncratic context of their organization.

## Conclusion

On the one hand, management theory is primarily concerned with providing conceptual order for managers that helps them deal with the complex, phenomenal world in which their organizations are evolving (Suddaby 2014). On the other hand, the scope of climate change goes beyond the organizational level that typically concerns management scholars and practitioners. Why, then, should management theorists consider climate change? Besides the obvious claim that no organization can thrive on a dying planet, addressing climate change in management theory may also serve the advancement of knowledge building more broadly. Indeed, in this article we have highlighted how several deficiencies in the conceptualization of the natural environment (NE) in management theory have hindered the development of actionable theoretical concepts and precluded crucial interdisciplinary collaboration and dialogue on the topic of climate change. Looking at how other fields have integrated and developed knowledge on the NE helped us to envision a way forward for management scholars and practitioners. Relying on tools from the philosophy of science we have highlighted several conditions for improving the representation of the NE in management theory.

We reiterate the claims from various organizations and management scholars that only relying on the environmental management perspective is an inadequate response in the face of the impending climate change catastrophe. We need to engage urgently with a broader conception of the NE; otherwise we run the risk of becoming irrelevant or playing second fiddle to other disciplines in this increasingly critical realm.

We believe that in order to be able to tackle the numerous challenges associated with climate change, management scholars and practitioners will need actionable theoretical concepts that integrate accurate knowledge on the NE. We thus proposed that a faithful epistemic representation of the NE be developed in order to allow both scholars and practitioners alike to perform new surrogative reasoning.

## Appendix

**Table:**

AMR Articles mentioning the NE or ecology (from most recently published)
Bansal, P., Kim, A. and Wood, M.O., 2018. Hidden in plain sight: The importance of scale in organizations’ attention to issues. *Academy of Management Review*, *43*(2), pp.217–241
Mikko, M., Saku, S. and Cornelissen, J., 2017. Reasoning by analogy and the progress of theory. *Academy of Management Review*, *42*(4), pp.637–658
Schrempf-Stirling, J., Palazzo, G. and Phillips, R.A., 2016. Historic corporate social responsibility. *Academy of Management Review*, *41*(4), pp.700–719
Mena, S., Rintamäki, J., Fleming, P. and Spicer, A., 2016. On the forgetting of corporate irresponsibility. *Academy of Management Review*, *41*(4), pp.720–738
Mitchell, R.K., Weaver, G.R., Agle, B.R., Bailey, A.D. and Carlson, J., 2016. Stakeholder agency and social welfare: Pluralism and decision making in the multi-objective corporation. *Academy of Management Review*, *41*(2), pp.252–275
Sonenshein, S., 2016. How corporations overcome issue illegitimacy and issue equivocality to address social welfare: The role of the social change agent. *Academy of Management Review*, *41*(2), pp.349–366
Spisak, B.R., O'Brien, M.J., Nicholson, N. and van Vugt, M., 2015. Niche construction and the evolution of leadership. *Academy of Management Review*, *40*(2), pp.291–306
Gray, B., Purdy, J.M. and Ansari, S., 2015. From interactions to institutions: Microprocesses of framing and mechanisms for the structuring of institutional fields. *Academy of Management Review*, *40*(1), pp.115–143
Hahn, T., Preuss, L., Pinkse, J. and Figge, F., 2014. Cognitive frames in corporate sustainability: Managerial sensemaking with paradoxical and business case frames. *Academy of Management Review*, *39*(4), pp.463–487
Wijen, F., 2014. Means versus ends in opaque institutional fields: Trading off compliance and achievement in sustainability standard adoption. *Academy of Management Review*, *39*(3), pp.302–323
Donaldson, T., 2012. The epistemic fault line in corporate governance. *Academy of Management Review*, *37*(2), pp.256–271
Smith, W.K. and Lewis, M.W., 2011. Toward a theory of paradox: A dynamic equilibrium model of organizing. *Academy of management Review*, *36*(2), pp.381–403
Bagley, C.E., 2008. Winning legally: The value of legal astuteness. *Academy of Management Review*, *33*(2), pp.378–390
Barnett, M.L., 2007. Stakeholder influence capacity and the variability of financial returns to corporate social responsibility. *Academy of management review*, *32*(3), pp.794–816
Aguilera, R.V., Rupp, D.E., Williams, C.A. and Ganapathi, J., 2007. Putting the S back in corporate social responsibility: A multilevel theory of social change in organizations. *Academy of management review*, *32*(3), pp.836–863
King, A., 2007. Cooperation between corporations and environmental groups: A transaction cost perspective. *Academy of Management Review*, *32*(3), pp.889–900
Den Hond, F. and De Bakker, F.G., 2007. Ideologically motivated activism: How activist groups influence corporate social change activities. *Academy of management review*, *32*(3), pp.901–924
Sirmon, D.G., Hitt, M.A. and Ireland, R.D., 2007. Managing firm resources in dynamic environments to create value: Looking inside the black box. *Academy of management review*, *32*(1), pp.273–292
Van Oosterhout, J., Heugens, P.P. and Kaptein, M., 2006. The internal morality of contracting: Advancing the contractualist endeavor in business ethics. *Academy of Management Review*, *31*(3), pp.521–539
Peredo, A.M. and Chrisman, J.J., 2006. Toward a theory of community-based enterprise. *Academy of management Review*, *31*(2), pp.309–328
Cornelissen, J., 2006. Metaphor in organization theory: Progress and the past. *Academy of Management Review*, *31*(2), pp.485–488
Godfrey, P.C., 2005. The relationship between corporate philanthropy and shareholder wealth: A risk management perspective. *Academy of management review*, *30*(4), pp.777–798
Aragón-Correa, J.A. and Sharma, S., 2003. A contingent resource-based view of proactive corporate environmental strategy. *Academy of management review*, *28*(1), pp.71–88
Newton, T.J., 2002. Creating the new ecological order? Elias and actor-network theory. *Academy of Management Review*, *27*(4), pp.523–540
Wade-Benzoni, K.A., Hoffman, A.J., Thompson, L.L., Moore, D.A., Gillespie, J.J. and Bazerman, M.H., 2002. Barriers to resolution in ideologically based negotiations: The role of values and institutions. *Academy of Management Review*, *27*(1), pp.41–57
Bazerman, M.H., Tenbrunsel, A.E. and Wade-Benzoni, K., 1998. Negotiating with yourself and losing: Making decisions with competing internal preferences. *Academy of Management Review*, *23*(2), pp.225–241
Barry, D. and Elmes, M., 1997. Strategy retold: Toward a narrative view of strategic discourse. *Academy of management review*, *22*(2), pp.429–452
Hanna, M.D., 1995. Environmentally responsible managerial behavior: is ecocentrism a prerequisite?. *Academy of Management Review*, *20*(4), pp.796–799
Shrivastava, P., 1995. The role of corporations in achieving ecological sustainability. *Academy of management review*, *20*(4), pp.936–960
King, A., 1995. Avoiding ecological surprise: Lessons from long-standing communities. *Academy of Management Review*, *20*(4), pp.961–985
Hart, S.L., 1995. A natural-resource-based view of the firm. *Academy of management review*, *20*(4), pp.986–1014
Jennings, P.D. and Zandbergen, P.A., 1995. Ecologically sustainable organizations: An institutional approach. *Academy of management review*, *20*(4), pp.1015–1052
Shrivastava, P., 1995. Ecocentric management for a risk society. *Academy of management review*, *20*(1), pp.118–137
Dutton, J.E. and Ashford, S.J., 1993. Selling issues to top management. *Academy of management review*, *18*(3), pp.397–428
Aktouf, O., 1992. Management and theories of organizations in the 1990s: Toward a critical radical humanism?. *Academy of Management Review*, *17*(3), pp.407–431
Sullivan, J.J., 1986. Human nature, organizations, and management theory. *Academy of Management Review*, *11*(3), pp.534–549
Brady, F.N., 1985. A Janus-headed model of ethical theory: Looking two ways at business/society issues. *Academy of Management Review*, *10*(3), pp.568–576
Maruyama, M., 1982. Mindscapes, management, business policy, and public policy. *Academy of Management Review*, *7*(4), pp.612–619
Smith, H.R. and Carroll, A.B., 1978. Is There anything “New” in management? A “Rip Van Winkle” Perspective. *Academy of Management Review*, *3*(3), pp.670–674
McFarland, D.E., 1977. Management, humanism, and society: The case for macromanagement theory. *Academy of Management Review*, *2*(4), pp.613–623
Aldrich, H. and Herker, D., 1977. Boundary spanning roles and organization structure. *Academy of management review*, *2*(2), pp.217–230
Post, J.E. and Epstein, M.J., 1977. Information systems for social reporting. *Academy of Management Review*, *2*(1), pp.81–87

## Footnotes

1 We reviewed systematically articles in the Academy of Management Review that contained the expression “natural environment” and/or the word “ecology.” We initially obtained 196 research articles, and then conducted a screening for removing the articles that did not include a relevant reference to either the NE or ecology in the biological sense of the term. For instance, we took out the articles that were related to “population ecology,” “organizational ecology,” and “social ecology.” In the appendix, we provide a list of the remaining 43 articles included in our analysis. We also searched for and reviewed conceptual articles from other top management journals (Administrative Science Quarterly, Management Science, Organization Science, Organization Studies, Strategic Management Journal). This additional research brought two other articles (i.e., Böhm, Misoczky, & Moog 2012; Chen 2001).

2 The authors tracked the number of publications on Google Scholar.

3 See https://www.ipbes.net/review-values-assessment-first-order-draft
